# Fish By-Product Collagen Extraction Using Different Methods and Their Application

**DOI:** 10.3390/md22020060

**Published:** 2024-01-24

**Authors:** Sunita Gaikwad, Mi Jeong Kim

**Affiliations:** 1 Interdisciplinary Program in Senior Human Ecology, Changwon National University, Changwon 51140, Republic of Korea; sunitaranjitgaikwad1993@gmail.com; 2 Department of Food and Nutrition, Changwon National University, Changwon 51140, Republic of Korea

**Keywords:** collagen, fish byproducts, extraction, biomedical source, food additive

## Abstract

The processing of fishery resources results in the production of a growing quantity of byproducts, including heads, skins, viscera, intestines, frames, and fillet cutoffs. These byproducts are either wasted or utilized for the production of low-value items and fish oil. Typically, fish processing industries use only 25%, while the remaining 75% is considered as waste by-products. This review presents a comprehensive review on the extraction of collagen from fish byproducts, highlighting numerous techniques including acid-soluble collagen (ASC), enzyme-soluble collagen (ESC), ultrasound extraction, deep eutectic solvent (DES) extraction, and supercritical fluid extraction (SFE). A detailed explanation of various extraction parameters such as time, temperature, solid to liquid (S/L) ratio, and solvent/pepsin concentration is provided, which needs to be considered to optimize the collagen yield. Moreover, this review extends its focus to a detailed investigation of fish collagen applications in the biomedical sector, food sector, and in cosmetics. The comprehensive review explaining the extraction methods, extraction parameters, and the diverse applications of fish collagen provides a basis for the complete understanding of the potential of fish-derived collagen. The review concludes with a discussion of the current research and a perspective on the future development in this research field.

## 1. Introduction

The utilization of biomaterials sourced from natural origins and their many functionalities has garnered significant interest due to their innate biocompatibility, biodegradability, and innovative functionalities [1]. Collagen, among the recognized biomaterials, has a significant and extensive track record as a critical product for several applications, including those in the cosmetic, food, biomedical, and pharmaceutical industries [2]. The most common applications of collagen derived from mammalian sources are medication delivery, gene delivery, wound treatment, fundamental matrices for cell culture [3], tissue engineering such as bone substitutes, and skin replacement of artificial blood valves and vessels [4,5]. Bovine and porcine are the primary sources of collagen, which are considered to be the best collagen sources, owing to their high sequence homology with human collagen [6]. Despite the fact that animals are the primary sources of collagen, it is essential to note that the animal-derived collagens are not without associated risks. The utilization of collagen derived from bovine and porcine sources has significant hazards to human well being due to the potential transmission of diseases through blood, such as bovine spongiform encephalopathy, foot and mouth disease (FMD), caprine scrapie, ovine, and other zoonoses [7,8,9]. Moreover, clinical observations have suggested that about 2.4% of people are allergic to collagen derived from porcine and bovine sources [10,11]. To overcome such health-related issues, the consumption of collagen derived from marine sources may provide a safe alternative for consumers [12].

Collagen is found in various marine organisms, such as fish (Pisces), jellyfish (Cnidaria), sponges (Porifera), mollusks (octopus, mussel, cuttlefish, and squid), and specific echinoderm species [13]. An inherent benefit of marine-derived collagen is its compatibility with religious beliefs as it avoids the use of bovine and porcine, which are not consumed among Hindus and Muslims, respectively. Global fish production reached 175 million tons in 2017 and is projected to reach over 194 million tons by 2026 [14]. Approximately 25% of the entire weight of the fish is utilized by the fish processing industry, while the remaining 75% is classified as waste by-products, including skeletons, bones, heads, viscera, tails, skins, and other discards from fisheries [15]. A total of 30% of the total waste by-products consist of scale, bone, and skin, which can be utilized to generate collagen due to their abundance of high-quality proteins [16]. Utilizing fish waste for collagen production has the potential to provide high-value goods and mitigate environmental issues, therefore bolstering the economic prosperity of fish processing industries [17,18]. Fish collagen has higher bioavailability and has approximately a 1.5 times greater absorption efficiency compared to its bovine- and porcine-derived alternative [19]. Despite these advantages, fish collagen presents some drawbacks, such as low mechanical strength, amino acid contents, biomechanical stiffness, and melting point, and has a high biodegradation rate [3].

**Figure 1:**
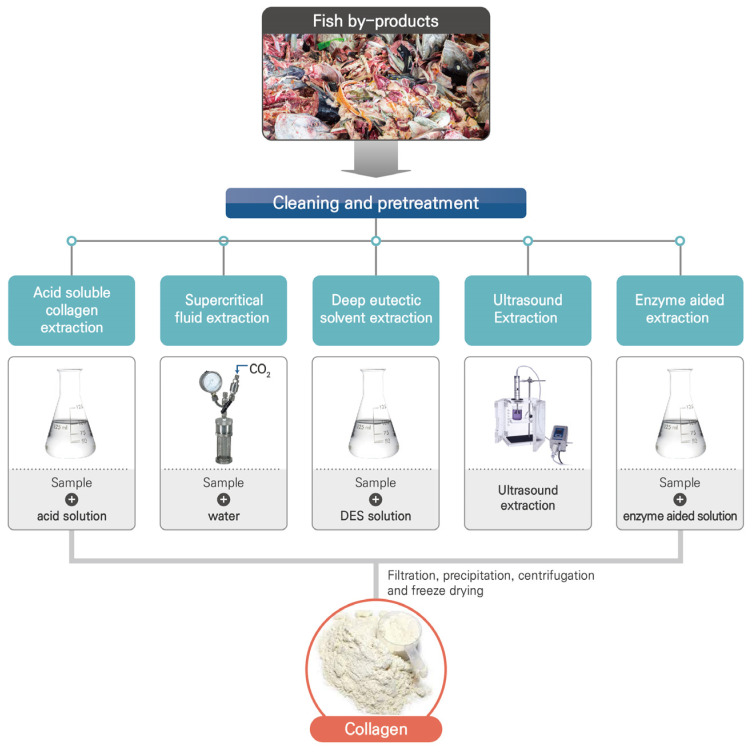
Collagen extraction using different methods. DES, deep eutectic solvent.

In addition to collagen, hydrolysates and their peptides have also gained significant attention as a functional ingredient owing to their various health benefits. Collagen peptides are fragments composed of more than two amino acid residues and exhibit physiological functionality on its consumption in vivo [20]. Generally, these peptides can be obtained using traditional techniques including fermentation, enzymatic hydrolysis, and microbial processes [21]. These peptides have various health benefits and can be used in the food sector as potential nutritional supplements. Various fish by-products such as bone, skin, muscle, and internal organs are used to isolate a number of bioactive materials, among which the fish intestine is a crude enzyme source, the bones are a source of calcium, and the remaining skin and protein are expensive materials for the isolation of bioactive peptides [22]. Lee et al. investigated the photoaging and antioxidative stress inhibition potential of a collagen peptide (Naticol^®^) against ultraviolet B (UVB) radiation using cell and animal models [23]. The study revealed that the collagen peptide Naticol^®^ improved skin moisturization through factors related to the hyaluronic acid ceramic synthesis in the cells (HaCaT) and hairless mice (SHK-I) that were exposed to the UVB radiation. Moreover, Naticol^®^ exhibited an inhibition ability against wrinkle formation in Hs27 cells and SHK-I exposed to UVB radiation and retained 3-isobutyl-1-methylxanthine-induced cells (B16F10) and SHK-I mice irradiated to the UVB [23]. Based on the findings of the study, it was concluded that the ingestion of Naticol^®^ might have potential to prevent skin photoaging. In another study, Cho et al. investigated the protective effect of a fish collagen peptide (FC) isolated from Oreochromis niloticus against skin photoaging [24]. The results demonstrated that the FC isolated from Oreochromis niloticus demonstrated resistance to skin dryness and wrinkle formation induced by ultraviolet B radiation, both in vitro and in vivo, through its antioxidant and anti-inflammatory properties. This review focuses on the latest developments in fish collagen-based biomaterials, including the extraction techniques employed (Figure 1) and their diverse applications across several domains.

## 2. Extraction Techniques of Collagen from Fish By-Products

The process of obtaining collagen from fish by-products involves several steps, namely preparation (which includes cleaning the raw material and applying chemical treatment), extraction, precipitation, and recovery. The first step includes washing, cleaning, separation of raw material, and cutting the samples into small pieces to facilitate pretreatment of the samples. In addition, the samples are pretreated to improve the effectiveness of the extraction process and eliminate components that are non-collagenous. The pretreatment process aims to remove non-collagenous materials and enriches the collagen. Moreover, the triple-helix structure in collagen makes its resistant to most proteases [25]. Thus, prior to extraction, pretreatment is performed using a weak acid or base to disintegrate the crosslinked collagen under controlled conditions such as acid concentration, exposure time, and temperature in order to disintegrate the cross-linked collagen. Also, it is necessary to demineralize the raw materials to increase the collagen extraction yields. Typically, collagens are present as triple-helix fibers that have stable hydrogen connections both between and within the molecules, which makes them insoluble in water. Therefore, it is imperative to employ appropriate extraction methods involving chemical solvents, enzymes, or extraction instruments in order to optimize the solubilization of collagen proteins and facilitate their isolation. Additionally, preservation of fish by-products is crucial in order to maintain the quality of extracted collagen. Adequate storage and quick processing help to preserve the biochemical and structural properties of the obtained collagen, which could prevent degradation and maintain its functional attributes. The individual extraction of collagen-rich byproducts including skins, bones, heads, scales, and viscera could produce good-quality collagen upon maintaining their integrity with adequate preservation.

### 2.1. Acid-Soluble Collagen (ASC) Extraction

Typically, fish collagen is extracted using solely acidic solutions in the ASC extraction process. The primary purpose of acid extraction is to disrupt the cross-links within the collagen helix, resulting in the formation of superior-quality collagen. This process also leads to the depolymerization of heavy-weight proteins into shorter peptides, which typically range in size from 0.3 to 8 kDa [26]. Hence, the ASC extraction method is used to examine the extraction efficiency by utilizing various acids, resulting in a high yield of collagen with maximized purity. Acetic acid (AcOH) is a commonly utilized acid in the process of extracting collagen from marine sources. The acid extraction solution has a concentration range of 0.5–1 M, which enables the breakdown of both intra- and inter-molecular cross-links while preserving the structure of the collagen chain. Recently, Kuwahara et al. conducted collagen extraction from tilapia scales with a yield of 1.58% by bubbling CO_2_ through 0.1 M of acetic acid for five hours [27]. Although acetic acid is commonly used in most studies, Tan et al. investigated the use of various acids (AcOH, citric acid, hydrochloric acid, and lactic acid) at pH 1.8 to 3.0 and a solid to liquid ratio of 1:50 *w*/*v* for acid extraction, HCl at pH 2.3 to 2.5, and a solid to liquid ratio of 1.15–1.50 *w*/*v* for homogenization-aided extraction; and HCl at pH 2.4 with a solid to liquid ratio ranging from 1.20 to 1.50 and a pepsin concentration 0.118 to 23.6 KU/g skin for extracting collagen from catfish skin using acid extraction, homogenization-aided extraction, and pepsin-aided extraction [28]. The homogenization- and pepsin-aided extraction methods achieved a maximum protein recovery rate of 64.19% at a pH of 2.4 with HCl. On the other hand, the acid extraction approach yielded the highest protein yield of 42.36% at pH 2.4 with HCl. The findings contradicted previous studies that found decreased collagen yields when employing HCl compared to AcOH, which may be attributable to the use of different concentrations of acids and a different pH. Arumugam et al. conducted a study to examine how different concentrations of AcOH (0.2–1.0 M) and NaCl (0.1–2.5 M) affect the collagen yield obtained from Sole fish skin [29]. The maximum yield of collagen achieved was 1.93% at the optimal concentrations of AcOH (0.5 M) and NaCl (1.9 M). Bhuimbar et al. conducted a study to examine the impact of different acids on acid-solubilized collagen isolated from the skin of *Centrolophus niger* (black ruff) fish by suspending the skin in 0.5 M solutions of acetic, lactic, citric, formic, and sulfuric acid [30]. The collagen yield was the highest with lactic acid at 45%, followed by formic (32%), tartaric (31%), citric (31%), and acetic (25%) acid. HCl and H_2_SO_4_ showed negligible extraction yields which were associated with their low efficiencies. The acid-solubilized collagen methods isolated from several research studies utilizing acetic acid from fish by-products are presented in Table 1. Using inorganic acids like hydrochloric acid and sulfuric acid could hydrolyze the protein molecule. The hydrolysis of the protein may result in breaking the structural peptide bonds, which ultimately results in molecular degradation of collagen and molecular weight reduction. Koochakzaei et al. investigated the effect of sulfuric acid on gelatin and the results revealed that the hydrolysis caused by sulfuric acid led to a decreased α-helix chain and enhanced random coil and β-sheet structures [31]. The peak deconvolution study suggested the occurrence of degradation while transforming the α-helix to random coil and β-sheet structures [31].

### 2.2. Enzyme-Soluble Collagen (ESC) Extraction

Enzyme-soluble collagen (ESC) is the primary method for collagen extraction and is a green biotechnology which could reduce solvent use and waste generation by accelerating the decomposition reaction without using organic solvents and toxic chemicals to generate substitutes. Enzymatic pretreatment, such as pepsin, has been used to break down the telopeptide ends in the collagen chains and make it easier to remove proteins from the remaining matrix [32,33]. Yu et al. reported that collagen could be extracted with a maximum yield of 84.85% by using optimum conditions, including a pepsin concentration of 1389 U/g, hydrolysis time of 8.67 h, and a solid to liquid ratio of 1.57 [34]. Collagen extraction using pepsin was attempted with various tissues (skins, bones, scales, fins, and swim bladders) of bighead carp [35]. The skin (60.3%) and swim bladder (59%) yielded similar amounts of collagen, which were significantly higher than those from the fins (5.1%), scales (2.7%), and bones (2.9%). The collagen yield might be different depending on the fish species, its habitat, and the specific pretreatment and extraction method used [36]. Junianto et al. investigated the impact of AcOH and enzyme (pepsin) extraction on the collagen yield from nilem fish skin [37]. The findings demonstrated that the highest yield was 6.18% when using the optimum concentrations of AcOH (0.7 M) and pepsin (1.0%) [37]. Table 1 presents a concise overview of the extraction results under various conditions and with diverse marine species. In a recent study, Lee et al. examined the influence of the enzymatic extraction method (1% Alcalase) on the antioxidant and physicochemical characteristics of collagen extracted from Alaska pollack skin [38]. Jia et al. conducted a study where they produced an enzyme hydrolysate from Alaska pollack skin using various enzymes including Neutrase, Flavourzyme, Alcalase, trypsin, Protamex, papain, and pepsin to determine the most effective enzymes and optimum conditions for hydrolysis [39]. Two studies have confirmed that collagen hydrolysate obtained from Alaska pollack skin, utilizing different enzyme extraction methods, has potent antioxidant properties. Wang et al. conducted further investigation into the antioxidant effects of scallop protein hydrolysates (SPHs) produced through enzymatic hydrolysis using Alcalase, pepsin, and dispase [40]. The SPH hydrolyzed by alcalase exhibits a significant capacity to scavenge free radicals, which can be attributed to its high content of amino acids (35.25%), which possess antioxidant properties, as well as its remarkable solubility.

**Table 1:** Summary of the fish collagen sources and experimental conditions used for fish collagen extraction using the acid-soluble collagen (ASC) and pepsin-solubilized collagen (PSC) methods.

Source of Collagen	Extraction Solvent	Extraction Conditions	Yield (%)	Ref.
Scales of tilapia	30.2 M AcOH (CO_2_ bubble)	Time = 5 h Gas flow = 3 L/min S/L = 1/40	1.58	[27]
Catfish skin	AcOH, HCl, citric acid and lactic acid	Time = 60 h T = 4 °C pH = 1.8–3.0	5–42.36	[28]
Sole fish skin	0.5 M AcOH	Time = 32 h T = 25 °C S/L = 1/9	19	[29]
Scales of seabass	0.5 M AcOH	Time = 48 h T = 4 °C S/L = 1/10	0.38	[41]
Grass carp skin	0.5 M AcOH	Time = 72 h T = 4 °C S/L = 1/40	90	[42]
Cod skin	0.5 M AcOH	Time = 72 h T = 4 °C S/L = 1/10	Not evaluated	[43]
Small-spotted catshark skin	0.5 M AcOH	Time = 34 h T = 25 °C	61.24	[33]
Skin of catla and rohu fish	0.5 M AcOH	Time = 48 h T = 4 °C S/L = 1/60	69 (catla) 65 (rohu)	[44]
Tilapia skin and scale	0.5 M AcOH	Time = 24 h T = 4 °C pH = 7	3.2 (scale) 27.2 (skin)	[45]
Golden pompano skin and bone	0.5 M AcOH	Time = 48 h T = 4 °C S/L = 1/40	21.81 (skin) 1.25 (bone)	[46]
Swim bladder of yellow tuna	0.5 M AcOH	Time = 48 h T = 4 °C S/L = 1/10	1.07	[47]
Skin of giant croaker	0.5 M AcOH	Time = 6–10 h T = 4 °C pH = 1–4 S/L = 1.45–1.65 Pepsin = 800–2400 U/g	84.85	[34]
Fins, scales, skins, bones, and swim bladders of bighead carp	0.5 M AcOH	Time = 36 h T = 4 °C S/L = 1/10 Pepsin = 0.1%	61.8 (fins) 58.1 (scales) 71 (skins) 57.1 (bone) 75.2 (swim bladders)	[35]
Nilem fish skin	0.5–0.9 M AcOH	T = 4 °C Pepsin = 0.5–1.5%	4.25–6.18	[37]
*Lophius litulon* skin	0.5 M AcOH	T = 4 °C Pepsin = 1–6%	Not evaluated	[48]
Tilapia skin	0.5 M AcOH	Time = 48 h T = 4 °C Pepsin = 0.5%	Not evaluated	[49]
Silver carp scales	0.5 M AcOH	Time = 10–60 h T = 4 °C S/L = 1/10–1/50 Pepsin = 1.5%	12.06	[50]
Cod swim bladder	0.5 M AcOH	Time = 3 days T = 25 °C S/L = 1/10 Pepsin = 10%	11.53	[51]
Thornback ray skin	0.2 M AcOH	Time = 18 h T = 4 °C S/L = 1/10 Pepsin = 5 g/g	30.16	[52]
Skin of Alaska pollack	Water	Time = 18 h T = 55 °C Shaking frequency = 100 rpm Alcalase = 1%	65.3	[38]
Skin of Alaska pollack	Water	Time = 8 h T = 55 °C pH = 6.0 S/L = 1/6 Protamex = 0.2%	85.95	[39]
Skin of Clown featherback (*Chitala ornata*)	0.5 M AcOH	Time = 18 h T = 4 °C S/L = 1/15 Ultrasound assisted Amplitude = 80% (10 min) Frequency = 20 kHz	57.35	[53]
Calipash of soft-shelled turtle	0.5 M AcOH	Time = 24 h T = 4 °C S/L = 1/20 Ultrasound assisted Ultrasonic power = 200 W Frequency = 24 kHz (24 min)	50.75	[54]
Skin of sea bass	0.1 M AcOH	T = 4 °C S/L = 1/200 Ultrasound assisted Amplitude = 80% (24 h) Frequency = 20 kHz	90.40	[55]
Skin of Atlantic cod fish	Deep eutectic solvent Composite of Urea (U) and lactic acid (LA)	U:LA ratio = 1.2 T = 4 °C S/L = 1/10 Time = 48 h	6	[56]
Skin of cod fish	Deep eutectic solvent Composite of Cholinium chloride (CC) and oxalic acid (OA)	CC:OA ratio = 1.1 Time = 2 h T = 65 °C	96.01 (extraction efficiency)	[26]
Skin of Atlantic cod fish	Supercritical fluids Water (CO_2_ bubble)	T = 37 °C Pressure = 50 bars S/L = 1/20 Time = 3 h	13.8	[57]

### 2.3. Ultrasound Extraction

Ultrasound is a promising method for extracting collagen, resulting in improved extraction efficiency. Ultrasound stimulates the formation of cavities in the solvent, resulting in the creation of microbubbles that cause the collapse of fish tissues and enhance the interaction between the solid and liquid phases. An increase in ultrasonic frequency leads to a reduction in bubble size and an increase in shear forces [54,58,59]. Petcharat et al. explored the ultrasound-assisted extraction of collagen from clown featherback skin by applying different amplitudes (20–80%) and times (10–30 min). In their study, the collagen yield increased significantly (*p* > 0.005) from 27.18 to 57.35% with the augment of ultrasonication time [53]. Furthermore, Zou et al. examined the influence of ultrasound on the collagen yield extracted from soft-shelled turtle calipash [54]. The optimal extraction conditions (200 W, 24 kHz, and 24 min) resulted in increases in collagen yield of 16.3% compared to traditional AcOH extraction [44]. In the study of Kim et al., collagen extraction using sea bass skin reached up to 90.40% with ultrasound treatment for 24 h in 0.1 M AcOH. The optimal ultrasound extraction conditions for collagen were found to be a frequency of 20 kHz, an amplitude of 80%, and a solution of 0.1 M acetic for a duration of 3 h [55]. Petcharat et al. investigated the effect of ultrasound treatment on collagen isolated from clown featherback (*Chitala ornata*) skin [53]. The results demonstrated increased collagen yield from 27.18–57.35% with utilization of ultrasound treatment. A thermal transition study suggested that ultrasound treatment at harsh conditions (80% amplitude and 30 min) resulted in protein cleavage, which led to the liberation of low-molecular-weight protein fractions. The liberation of low-MW protein fractions may result in low enthalpy [53].

### 2.4. Deep Eutectic Solvent (DES) Extraction

The deep eutectic solvent (DES) method could be employed as an environmentally friendly approach for collagen extraction from plant, animal, and marine sources, involving the formation of strong hydrogen bond interactions comprising at least one hydrogen bond acceptor (HBA) and a hydrogen bond donor (HBD) [60]. The incorporation of water into the DES for extraction is a widely recognized approach that enhances mass transfer processes by reducing viscosity [61,62]. Bisht et al. extracted collagen from Atlantic cod fish by utilizing aqueous solutions of different DESs consisting of urea (U), betaine (Bet), and cholinium chloride (CC) as the HBA and acetic acid, lactic acid, and propionic acid as the HBD [56]. The highest extraction yield of collagen was 6% at the optimal aqueous solution of urea: lactic acid (1:2) at a concentration of 0.75 M. In addition, Bai et al. reported that the extraction efficiency of collagen from cod skin, using a DES consisting of choline chloride (CC) and oxalic acid (OA), was 90% and they examined the impact of temperature on CC: OA extraction yield [26]. In their study, they found that higher temperatures increased collagen extraction due to a decreased viscosity and increased mass transfer coefficient [26]. In conclusion, elevated temperatures not only contribute to the purification of extracted collagen but also promote collagen polymerization above 55 °C, resulting in a maximum efficiency of 96.01% at 65 °C. Batista et al. reported the green extraction of collagen from blue shark skin using a natural deep eutectic solvent composed of citric acid: xylitol: water (1:1:10 molar ratio) [63]. The isolated collagen was identified as type I collagen with a 2.5 times higher collagen yield compared to the conventional one and an exceptionally reduced extraction time from 96 to 1 h. The in vitro cytotoxicity study revealed the biocompatibility of the extracted material.

### 2.5. Supercritical Fluid Extraction (SFE)

Supercritical fluid extraction (SFE) is a popular green extraction technique with various advantages compared to commercial extraction methods, including more selectivity, improved fractionation capabilities, reduced environmental impact, and a higher collagen yield, and can be used to extract high-value compounds from natural products and food with increasing reliability in the sector of heavy metal recovery and drug delivery [64]. The SEF method relies on the solvent’s solvating characteristics, utilizing pressure and temperature beyond the solvent’s critical point to induce substantial physical alterations that enhance the solvent’s capabilities [65]. In SEF, CO_2_ is a commonly used molecule due to its exceptional properties such as low toxicity, cost-effectivity, stability, high availability, flammability, environment acceptability, and mild operating conditions. In a recent study, this method was used to extract collagen from cod skin, resulting in a maximum yield of 13.8% [57]. Specifically, the skins were placed in a high-pressure vessel with distilled water and pressurized with CO_2_ gas up to 50 bars. The obtained collagen could be used in the cosmetic, biomedical, and pharmaceuticals industries, with clear economic advantages [57].

The acid-soluble collagen extraction method is a simple and versatile method of extraction which provides a high collagen yield. However, this method involves the use of relatively hazardous chemicals, which makes it less ecofriendly. Additionally, the time required for ASC extraction is comparatively high. Regarding enzyme-soluble collagen (ESC) extraction, it is an ecofriendly method which reduces the use of toxic solvents. However, the cost of the enzymes may be a point of concern regarding the implementation of this method in industrial applications. Ultrasound extraction, deep eutectic solvent extraction, and supercritical fluid extraction are the methods most known for their environmentally friendly approach to collagen extraction, which produces maximum yield in a reduced time period. The ultrasounds and supercritical fluid extraction, both associated with high use costs, may lead to concerns with their application in large-scale extractions. In the case of deep eutectic solvent extraction, the optimization of conditions including DES composition and extraction parameters is a major challenge.

## 3. Collagen Extraction Parameters

In addition to extraction parameters, intrinsic characteristics of the biomass such as the fish species, body part or tissue used, original collagen content, and the age of the individual may influence the yield and quality of the collagen. The collagen extraction yields from marine sources are influenced by the time, temperature, solvent to liquid ratio, and solvent concentration. The impact of various parameters might be assessed and modified to determine the appropriate ranges for achieving optimal collagen isolation.

### 3.1. Time

Collagen extraction experiments are mediated by the diffusion process and explained by the rate of mass transfer. Due to the time-dependent nature of the diffusion process, the extraction yield will progressively increase as the extraction duration is extended. Alfaro et al. investigated various parameters including temperature, concentration of H_2_SO_4_ and NaOH, and duration to determine their impact on the yield of collagen [66]. Prolonged extraction periods could result in peptide degradation. Moreover, lengthy extraction procedures will make the technique less suitable for industrial applications. For example, Arumugam et al. extended the time of collagen extraction from sole fish skin and reported that the optimum time was from 12 to 60 h [29]. The highest collagen yield (19.18 mg/g) was recorded at 36 h but prolonging the extraction period resulted in a decline in yield, likely due to the breakdown of the collagen, which may be attributed to the degradation of the collagen chain caused by acetic acid. During the extraction process, an increase in temperature decreases the viscosity of the solution, leading to a higher rate of mass transfer [29].

### 3.2. Temperature

The temperatures required for collagen extraction differ among fish species. As represented in Table 1, the optimal temperature range for extracting collagen from fish is 4–25 °C. This is because the denaturation of collagen derived from most fish species begins at temperatures between 25 and 30 °C [59]. Nevertheless, previous studies suggested the fish collagen can denature at temperatures lower than 20 °C as well. For instance, collagen has been isolated from the skin of codfish denatured at around 16 °C, which could be attributed to the habitat of the species [43]. In another study, a denaturation temperature of 15.2 °C was reported for collagen isolated from Baltic cod skin [2] Nevertheless, pepsin-aided collagen extraction requires lower temperatures (4–10 °C) due to pepsin’s susceptibility to elevated temperatures [67]. The use of pepsin to extract collagen at high temperatures might cause self-digestion, deactivation, and an increase in temperature beyond the denaturation point, resulting in the breakdown of proteins and rendering pepsin ineffective. The collagen extraction procedure from the fish sources typically take place at 4 to 10 °C, where pepsin is able to cut the telopeptides which facilitate their solubilization in an acid solution [67].

### 3.3. Solvent/Pepsin Concentration

ASC extraction could cause peptide degradation, leading to a decrease in both the purity and yield of the extracted collagen, as a result of the increase in solvent concentration. A previous study evaluated the effect of solvent concentration (specifically acetic acid) on the extraction efficiency of sole fish skin [29]. The concentration was within the range of 0.2 to 1 M [29]. The highest amount of collagen yield was 15.97 mg/g at an acetic acid concentration of 0.6 M. With a further increase in concentration, the collagen yield decreased to 12.5 mg/g. The decrease in collagen yield might be associated with the degradation of collagen peptides due the excessive acid concentration. In the PSC extraction method, pepsin is used to cleave the cross-link of the collagen triple-helix. Improved enzyme concentration can increase the digestion rate of telopeptides and consequently increase extraction efficiency. However, all telopeptide ends undergo cleavage at the threshold value of pepsin (10% concentration, may vary with nature of species). Pepsin addition exceeding the threshold value can degrade the solubilized collagen and could, in turn, decrease the collagen yield.

## 4. Applications

### 4.1. Biomedical Applications

#### 4.1.1. Wound Healing

Wound healing involves various stages, including coagulation, granulation, inflammation, proliferation, fibrogenesis, wound contraction, matrix synthesis, and deposition, which present challenges for the wound healing process. Typically, the administration of synthetic medications in burn victim cases could potentially result in the development of drug resistance. In order to overcome such obstacles, it is imperative to formulate pharmaceuticals derived from natural sources. According to most studies, collagen hydrogels are considered the optimal and most dependable wound dressings due to their ability to self-arrange and cross-link, resulting in the formation of very stable and strong fibers [68]. There are several commercially available skin substitutes made from collagen that come from different sources, such as xenogeneic and allogeneic tissues, including Alloderm™, Matriderm™, Permacol™, Biobrane™ and Integra™ [69]. The elasticity and flexibility of the collagen fibers allow them to conform well to underlying topology in order to reduce healing time and pain, with improved long-term aesthetics. Collagen is involved in the development and migration of cells, tissues, and in extracellular matrix (ECM) formation. Furthermore, fish collagen-derived membranes exhibit high potential for medical applications, which may be associated with their porous and interconnected pore structure, ability to allow nutrients and oxygen to pass across the membrane, high-density cell seeding, and reduced immune response compared to other biomaterials derived from natural sources [70]. In addition to these characteristics, fish collagen has the ability to stimulate and promote the creation of fibroblasts and keratinocytes in the vicinity of the wound, leading to a notable enhancement in the healing process [68,71].

Elbialy et al. reported the improvement of cutaneous wound healing in rats using collagen derived from Nile tilapia skin [72]. The study concluded that applying tilapia-derived collagen to the stimulated wound healing and increased fibroblast and keratinocyte proliferation, ECM formation, and myofibroblast differentiation [72]. Furthermore, Shalaby et al. investigated the effect of collagen derived from tilapia and grey mullet fish on wound healing in rats, and the wound healing improved with increasing cell adhesion, which resulted in wound resolution and closure [72]. In a recent study, Pal et al. proved fish collagen’s potentiality as a skin substitute in full-thickness wound healing [73]. Additionally, the study revealed that wound healing is accelerated by the collagen sponge in a rat model. In another study, Hu et al. examined the wound healing activity of collagen peptides derived from nile tilapia skin in in vitro and in vivo assays [74]. Both in vitro and in vivo assays revealed that the nile tilapia-derived collagen peptides accelerated the wound healing process [74]. Table 2 summarizes recent studies regarding fish collagen’s applications in wound healing.

#### 4.1.2. Tissue Engineering

Collagen derived from fish has been widely employed in numerous tissue engineering applications, including the regeneration of skin, bone, and cartilage. Tissue engineering aims to regenerate damaged, eliminated, or diseased organs and tissues by utilizing porous and biocompatible scaffolds [82,83]. Possible causes of bone defects include infection skeletal abnormalities, tumor resection, trauma, atrophic nonunion, avascular necrosis, and osteoporosis [82,83]. Several techniques, such as allograft implantation, free fibula vascularized graft, the use of osteoprogenitor cells, and the application of osteoconductive scaffolds, have been developed to improve the process of bone regeneration [84,85]. Elango et al. conducted a study on the physico-functional and mechanical properties of collagen scaffolds in relation to their biocompatibility and ability to promote bone formation [86]. Three collagen-based scaffolds, namely collagen, collagen–hydroxyapatite, and collagen–chitosan were created, utilizing collagen sourced from blue shark cartilage. Interestingly, the rigidity (11–23 MPa) and rate of degradation of scaffolds were augmented with the incorporation of chitosan and hydroxyapatite [86]. The osteogenesis study indicated that the addition of both chitosan and hydroxyapatite to the collagen scaffolds significantly promoted the formation of osteoblast cells [86]. Moreover, the alkaline phosphatase (ALP) activity was higher in the osteoblast cells cultured in the collagen–hydroxyapatite scaffold, suggesting enhanced bone healing [86]. Skin defects can be a result of trauma, burns, genetic defects, scarring, and other diseases. To treat skin defects, two of the main strategies used are (1) wound dressing and (2) skin regeneration engineering. Zhou et al. prepared a nanofiber wound dressing from tilapia skin using electrospinning [87]. The proliferation, adhesion, and differentiation of human keratinocytes were enhanced by these tilapia collagen dressings, which could also facilitate rat skin regeneration. Cartilage tissue engineering has been employed for the purpose of repairing or regenerating articular cartilage that has been affected by disease or injury. In addition, collagen generated from fish shows promise in various tissue engineering applications, including those related to blood vessels [88], teeth [89], and the corneal [90]. A summary of recent studies on the utilization of fish collagen-based biomaterials and their composites in different tissue engineering applications can be found in Table 3.

#### 4.1.3. Drug Delivery

Extensive research has been conducted on fish collagen regarding its potential use in tissue engineering and wound dressings. However, its potential for drug delivery applications has not been thoroughly explored. Recent research has focused on the administration of drugs to particular organs in order to address issues such as inadequate stability, absorption, solubility, and bioavailability [108]. Wang et al. fabricated ferrous gluconate-loaded hydrogel microneedles from fish scale-derived collagen using a low-temperature press method [109]. In the phosphate buffer, microneedles expanded to 340% of the initial weight and released 34.5% of the total drug contents during a 24 h period. Cao et al. investigated the role of a fish collagen/chitosan/chondroitin sulfate composite incorporated with basic fibroblast growth factor (bFGF)-loaded poly (lactic-co-glycolic acid) (PLGA) microspheres for use in drug delivery systems [110]. The investigation suggested that the spherical microspheres uniformly distributed into the scaffold pores and showed higher biocompatibility compared to the scaffold with no microspheres. In addition, the scaffold microspheres showed an increased protein release and high diffusion rate, which may be attributed to the high rate of degradation and swelling. These results support the use of scaffold microspheres in tissue engineering applications. Nguyen et al. developed a fish scale-derived collagen hydrogel mixed with carrageenan to enhance the biocompatibility of allopurinol, a medicine used to treat gout and high levels of uric acid in the body [111]. Their study found that the resulting hydrogel increased the physical and bioactive properties of the drug in human body fluid, and the drug released by the carrier was approximately 1.5–6.7 times slower compared to the control sample [111].

#### 4.1.4. Cell Culture

The high biocompatibility of fish-derived collagen makes it a promising alternative compared to traditional mammalian collagens in cell culture applications. For example, tilapia type I atelocollagen was investigated for the differentiation and proliferation of preosteoblast MC3T3-E1 cells [112]. The mRNA expression of the 10 evaluated genes involved in the differentiation and proliferation increased after 3 days [89]. Additionally, gene expressions of the alkaline phosphatase, bone sialoprotein, and osteocalcin increased, while that of osteopontin remained unchanged. The 3D cell culture of the scaffold prepared from the fish collagen (FC) and polycaprolactone (PCL) was evaluated by Choi et al. [113], using electrospinning for the fabrication of the scaffold. The FC/PCL scaffolds promoted adhesion, protrusions, and proliferation. Furthermore, the scaffold that was constructed induced gene and protein expression [113]. The results indicate that the FC/PCL scaffold has potential applications in 3D cell culture for TECs [113]. A further investigation examined the capacity of collagen generated from tilapia fish to induce osteoblastic development in human mesenchymal stem cells (hMSCs) [114]. The hMSCs were pre-cultured on dishes coated with tilapia collagen and porcine collagen and attached to the collagen derived from tilapia scales, exhibiting a notable increase in the acceleration of osteoblastic differentiation throughout the early stages of the in vitro cell cultures [114]. The research studies described above discuss the potential of collagen materials obtained from fish in cell culture applications.

### 4.2. Food Sector

In modern society, the body experiences a decline in collagen synthesis as a result of improper dietary habits and the natural process of aging. As a result, collagen has become a demanded ingredient in the development of healthy food. Consuming collagen through dietary means is the optimal substitute for collagen injections. To meet the requirement for collagen ingestion, it is commonly combined with various types of foods.

#### 4.2.1. Collagen Supplements

The positive effects on health attributed to collagen have led to the creation of factories dedicated to producing collagen supplements. With increasing age, collagen synthesis in the body decreases, which makes tissues weaker and thinner. Collagen metabolism can assemble bone, ligaments, and skin and attracts fibroblasts, which, in turn, develops collagen fibrils in the cohesion and dermis of the collagen fibers. Therefore, tissue thickness, resilience, and hydration is increased [115]. Collagen supplements can decrease injury recovery time, boost muscle gain, and reconstruct damaged joints [116]. Type II collagen is effective in the treatment of chronic inflammatory sickness, rheumatoid arthritis, and the swelling and stiffness of multiple joints [116].

#### 4.2.2. Collagen as Food Additives

Food additives refers to substances added to food while processing in order to improve the taste, color, or flavor. Collagen and its derivatives are utilized as a food additive owing to their molecular structure, with hydrophilic and hydrophobic groups that present protein side chains. These properties contribute to their emulsifying, film forming, and foaming properties [117]. Moreover, fish collagen has also been studied as nutritional and functional food ingredients to improve the protein content of food products [118,119]. Collagen, a substance used in food additives, has been studied for its potential as an emulsifier in food applications [120]. One study evaluated the emulsifying properties of collagen fibers in an oil–water emulsion under a range of different pHs, protein concentrations, and devices used for emulsification [120]. The results showed that with a decrease in protein concentration and pH, the phase separation and droplet size of the emulsion decreased, allowing for the production of electrostatically stable emulsions at an acidic pH of 3.5 [120]. These emulsions showed shear-thinning behavior and a lower viscosity and consistency index at high homogenization pressures [120]. The researchers suggest that fish collagen could be used as an emulsifier in the food sector, particularly in acidic products [120]. Bhagwat et al. investigated the effect of collagen isolated from carp (Cyprinus carpio) by its incorporation into the milk-based food product paneer [8]. The results suggested that the paneer developed with fish scale-derived collagen demonstrated a good sensorial and textural attribute. In another study, Kumar et al. prepared dietary biscuits with marine collagen peptides added and studied the physical, sensorial, and textural behavior of the biscuits [121]. The addition of collagen peptides resulted in enhanced protein and antioxidant potential. The as-prepared biscuits were slightly darker and sensorially preferred by the sensory panelists, with the remark ‘liked moderately-very much’. Kittiphattanabawon et al. investigated the effect of a blacktip shark skin hydrolysate added to linoleate and cooked pork and the results demonstrated its ability to inhibit lipid oxidation [122]. In the same context, Jridi et al. reported that the addition of a gelatin hydrolysate isolated from Alcalase cuttlefish skin into turkey meat sausage (0.5 mg/g) resulted in delayed lipid oxidation [123].

#### 4.2.3. Collagen in Drinks

Many collagen-based drinks such as cocoa, juice and soy and a bird’s nest drink with collagen have been released by various manufacturers. Usually, collagen drinks stimulate the collagen formation mechanism in the consumer’s body, leading to reduced skin wrinkles and sagging. Avon has manufactured the ‘Avon Life Marine Fish Peptide Collagen Drink’ prepared using Salmon fish skin-derived collagen, vitamin C, and fructo-oligosaccharides [124]. Moreover, Nestle Malaysia has manufactured fish-derived collagen containing Nescafe Body Partner, Collagen Coffee, and Kacip Fatimah. The collagen has a characteristic caprylic taste. However, the taste of collagen can be improved by blending sucralose and stevia extract, followed by further blending with acesulfame potassium [125].

### 4.3. Collagen in Cosmetics

Collagen, in particular, is the primary constituent of skin, with natural polymers playing a significant role in this. The production of collagen in human skin is carried out by fibroblasts, which synthesize procollagen that is then transformed into collagen molecules [126,127,128,129]. Additional studies demonstrate the advantageous impacts of collagen and collagen peptides in enhancing skin characteristics due to their antibacterial, anti-wrinkling, and anti-aging qualities. Furthermore, collagen peptides include antioxidant properties that aid in safeguarding the skin against free radicals and counteracting their aging effects [126]. Borumand et al. documented that an oral drink containing hydrolyzed collagen enhanced the depth of facial wrinkles and augmented skin elasticity [127]. Asserin et al. examined the impact of oral collagen peptide supplementation on skin moisture [129]. Oral supplementation of a collagen peptide increased skin moisture levels by 12% and indicated potential improvements in signs of skin aging [129]. Various studies have been conducted by cosmetic firms to explore the cosmetic uses of collagen, and consequently, there is a number of published materials accessible to the public that specifically address the use of collagen in cosmetics. Lu et al. conducted an in vivo study to examine the efficacy of collagen derived from the cartilage of blue sharks as a cosmetic on human wrist skin [130]. The skin properties test was evaluated by applying the cartilage gel containing 0.125 to 5% of lyophilized hydrolysate shark cartilage (LHSC) on the inner wrist skin for 10 and 20 min. Skin properties such as moisture level, texture level, complexion level, and oil level were examined using a skin analyzer. The results suggested that the addition of LHSC led to a significant improvement in texture, moisture, and complexion of the skin, while controlling the oil level with a wrinkle-smoothing effect. In another study, Martins et al. investigated the addition of green halibut skin collagen to the formulation of a cosmetic hydrogel [131]. The results revealed that a cosmetic hydrogel incorporated with fish collagen met criterion A of ISO 11930:2019 [132] related to the efficiency of the preservative system. Moreover, clinical testing of a cosmetic formulation containing marine collagen at concentrations of 0.1 to 0.5% was carried out on 23 healthy volunteers. The results showed a noticeable hydrating effect, indicating collagen’s potential application in cosmetics.

## 5. Conclusions and Future Perspective

By-products generated from fish processing industries are abundant, economically useful, and substantial sources of collagen on a broad scale. The exceptional biocompatibility and biodegradability of collagen generated from fish waste make it suitable for a wide range of applications. Moreover, collagen extracted from fish presents fewer or no issues regarding its use by people from various religions, compared to that obtained from porcine and bovine sources. In light of the growing demand for collagen, it is imperative to identify sustainable and economically viable sources of collagen.

This review provides a comprehensive overview of fish collagen sources and several extraction techniques, including acid-soluble collagen, pepsin-soluble collagen, ultrasound-assisted extraction, deep eutectic solvent extraction, and supercritical fluid extraction. Subsequently, an examination was conducted on the influence of different extraction parameters, including time, temperature, solid to liquid ratio, and solvent/pepsin on the quality and quantity of collagen obtained. In addition, the utilization of fish collagen and collagen-based biomaterials in biomedical contexts, including wound healing, tissue engineering, drug delivery, and cell cultures, was discussed. Furthermore, the utilization of collagen-based materials in cosmetic industries and the food sector was also highlighted.

Although marine sources of collagen are more appealing, there are still obstacles in their use due to their low melting and denaturation temperatures. Therefore, future studies should prioritize the advancement of knowledge on the physicochemical properties of collagen. We also call for an investigation into different combination strategies as the utilization of individual polymers is insufficient to meet the diverse requirements of different applications. To manufacture suitable collagen from fish sources, it is necessary to explore novel alterations in the physical, chemical, and enzymatic characteristics of the structure of fish collagen. By doing so, potential marine sources can be revealed which can overcome current challenges and may ultimately contribute to the progress in versatile fish collagen-based composite materials in various sectors.

## Figures and Tables

**Table 2:** Fish collagen-based biomaterials for wound healing applications.

Fish Species	Source of Collagen	Remarks	Ref.
Nile tilapia (*Oreochromis niloticus* L.)	Skin	Collagen extraction can increase b-fibroblast, TGF-β1 growth factor (b-FGF), fibroblast and myofibroblast proliferation, α-smooth muscle actin (α-SMA) gene expression, and tricellularar matrix ECM production.	[72]
Tilapia and grey mullet	Scale	Extracted collagen converted to stable, high-strength fibers via self-aggregation and cross-linking, which assist optimal moisture level maintenance at the wound site, accelerating wound healing.	[68]
*Melanogrammus aeglefinus*	Skin	Fish skin-derived collagen on mice formed fibrin, which resulted in reduced clotting time, faster epithelialization, and shorter wound healing time.	[75]
*Prionace glauca*, *Scyliorhinus canicula*, *Xiphias gladius*, and *Thunnus albacares*	Skin	Collagen extraction significantly accelerated the wound healing of second-degree burns and generation of new skin appendage.	[76]
Snakehead fish (*Channa striata*)	Skin	New dressing for burn healing that has the potential to be used by cross-linking biopolymer collagen with alginate to form functional group—CONH.	[77]
Tilapia	Skin	Animal experiments indicated that collagen/bioactive glass (Col/BG) composites can accelerate rat skin wound healing. In addition, the Col/BG nanofibers promoted the proliferation, adhesion, and migration of human keratinocytes.	[78]
Salmon	Skin	The WST-1 assay reveled that the fibroblasts were well proliferated in the collagen and elastin sponge (CES). Grafting the CES and Terudermis (traditional collagen sponge) on the skin defects of rats revealed no significant difference observed between the CES and Terudermis.	[79]
*Rachycentrn canadum* (cobia)	Skin	Chitosan–collagen–starch membranes (CCSMs) loaded with *Punica granatum* extract showed reduced wound surface area and enhanced epithelial cell proliferation with no scar after wound healing.	[80]
Catla	Scale	Ex vivo permeation studies showed that the formulate (curcumin-loaded fish scale collagen)–hydroxypropyl methyl cellulose (HPMC K100) nanogel exhibited high concentrations and a low irritation score, confirming the prepared formulate’s function in wound healing.	[81]

**Table 3:** Fish collagen-based scaffolds for tissue engineering applications.

Fish Species	Source of Collagen	Applications	Ref.
Tilapia	Scales	Corneal tissue engineering	[90]
Shark	Cartilage	Bone tissue engineering	[91]
*Lates calcarifer*	Scales	Corneal tissue engineering	[92]
Salmon	Skin	Bone tissue engineering	[93]
*Lates calcarifer*	Scales	Bone tissue engineering and bone implant	[94]
Salmon	Skin	Bone tissue engineering	[95]
Flat fish (*Paralichthys olivaceus*)	Skin	Bone tissue engineering	[96]
Tilapia	Scales	Skin tissue engineering and regeneration	[97]
Tilapia	Scales	Dental tissue engineering	[89]
African catfish, salmon, and Baltic cod	Skin	Tissue engineering	[2]
Flatfish (*P. olivaceus*)	Skin	Skin tissue engineering	[70]
Haddock	Skin	Tissue engineering	[98]
Fresh tilapia	Scales	Artificial cornea	[99]
Salmon	Skin	Bone and cartilage tissue engineering	[95]
Fish	-	Bone tissue engineering	[100]
Lates calcarifer	Scales	Bone tissue engineering	[101]
Salmon—*S. salar* and African catfish—*C. gariepinu*	-	Tissue engineering	[2]
Arothron stellatus fish	-	Skin tissue engineering	[102]
Larimichthys crocea	Scales	Skin tissue engineering	[103]
Shark	Skin	Bone tissue engineering	[104]
Blue shark	Skin	Cartilage tissue engineering	[105]
Tilapia	Skin	Cartilage tissue engineering	[106]
North Atlantic cod	Skin	Skin tissue engineering	[107]
Larimichthys crocea	Scale	Skin tissue engineering	[103]

## Data Availability

Not applicable.
